# In conversation with…John Wendell Severinghaus

**DOI:** 10.1186/s13728-015-0028-7

**Published:** 2015-06-30

**Authors:** Ned Gilbert-Kawai

**Affiliations:** UCL Centre for Altitude, Space and Extreme Environment Medicine, Institute of Sport and Exercise Health, 170 Tottenham Court Road, London, W1T 7HA UK

*Whilst attending a conference last year, I was fortunate enough to be seated at lunch next to a very eminent octogenarian academic. Conversation ensued, and I was instantly captivated by his stories. These were not narratives filled with scientific facts, but rather anecdotes of his life and the pathways he had taken to get where he was today. Fascinated by such accounts, I set about the task of interviewing persons of scientific acclaim, to learn more about their life stories and unwritten tales. ‘In conversation with…’ therefore offers readers a chance to share an abridged version of these conversations.*

*(In view of the need to condense three hours of conversation, the original interview text has been abridged, and then reviewed, revised, and approved by JWS.)*

**“I think that my father concluded that I was never going to be academic. I was too much of a gadgeteer. I loved playing with things, taking gadgets apart, and usually ended up putting them back together without a few unimportant screws or parts.”**

## Q. You were born in Madison, Wisconsin, in 1922. What did your parents do?

My mother was a great role model, a leader in many causes, and an expert gardener. My father was kind and generous to all throughout his life to 1986. He was a chemist at the University of Wisconsin who spent World War I trying to figure out antidotes for the war gases. Thereafter, he attended Harvard Medical School where he was headed for a career in research, as he wasn’t at all interested in becoming a practising doctor. In 1922, when insulin was discovered, he was the only MD with physiological chemistry knowledge regarding the chemistry of sugars on the basic science faculty in Madison. Consequently, while he had never taken an internship, he took charge of a new department taking care of diabetics, but also pituitary and thyroid patients. He took the opportunity and ran. Within a few years, he had become the United States’ most distinguished endocrinologist, president of the Endocrine Society, and editor of their annual yearbook. In 1941, just before World War II, Roosevelt invited him to be America’s first good will ambassador to South America to an international endocrinology meeting in Montevideo. He led the first Unitarian-Congregational relief mission to Europe under UNRRA after World War II. After a decade as director of clinical research at Hoffman LaRoche, he became professor of nutrition at Columbia University Medical School.

## Q. Do you believe it was his ambition and personal achievements that drove you to do all that you have accomplished?

My father made sure I received a different kind of education than most kids would have expected. He put me through courses in manual or practical training…and he made sure I did them. When I was about five or six, he gave me a wonderful book about making electrical things yourself. In 1927, there was not much radio around, so I started building crystal radios from this book. I guess it was probably this book that got me into studying and developing an interest in physics.

## Q. An interest which continued, for I believe physics was your first degree?

Yes, it was my first completed degree. I studied physics at Haverford College, a small Quaker school in the suburbs of Philadelphia. I had started out taking chemistry. Unfortunately, I did not think much of the chemistry teachers, but happened to note how wonderful the physics teachers were so I changed my major plan. In fact, while there, I got a job setting up the demonstrations for the physics faculty. They paid me to help them set up experimental apparatus before every lecture. That was a lot of fun.

## Q. How did you end up in medical school?

In 1942, World War II broke out and everybody of draft age was getting drafted. I, however, was one of three or four physics majors, so was told in no uncertain terms that after graduation I was to go to MIT Radiation Laboratory to work on RADAR. I had never heard of it at the time. After two years at MIT, on August 6, 1945, news reached me that an atom bomb had incinerated hundreds of thousands of Japanese residents of Hiroshima. This had a profound effect on me. Realising that ‘I’m a physicist, and people like me are responsible for the deaths of hundreds of thousands people with a single bomb’, I made a decision there and then to leave physics behind me. So what could I do? At the time I thought bio-physics involved putting electronics to use in physiology and medicine, so I called my father and said, ‘I think I’ll go to medical school. Can I immediately get into Wisconsin medical school?’ As it happened, he was good friends with the Dean, and half an hour later he called back and said ‘You’re admitted.’ This was because more than half the incoming medical students suddenly lost their military educational support.

## Q. There you met your wife, Elinor?

I met Elinor when I was at MIT. I had been sent to a student Christian movement meeting by the Congregationalist church where I attended. On arriving late, I was the only man and I was introduced to a circle of girls from New England colleges. As I was being introduced to Elinor Peck, a voice in my head said, ‘My God! That’s the woman I’m going to marry.’ Following our marriage five years later, we settled down in up-state New York Bassett Hospital, in Cooperstown (part of Colombia University), for a 2-year rotating internship.

## Q. It was at this time, whilst still a medical student, that you started your career in research?

Well, yes. I designed, built, and published an electrophrenic respirator based on an idea by Sarnoff at Harvard. The Borden Award was to be given for the best medical student research project. I hurriedly wrote a paper about the device, with diagrams and photos, and submitted it just in time. Fortunately, I won the award of $500. I realised I was not a businessman, for I had sold a number of these devices to various anaesthetic departments for eighty dollars when I could have charged eight hundred! The Borden award, and the money earned covered the cost of a delayed second honeymoon to Elinor’s parents in Guatemala.

Additionally, as a 2-year intern, I had 6 months in research with a fine internist-laboratory director, Joseph Ferrebee. In that time, I generated and published a paper on a method to use flame photometry for blood calcium measurement.

## Q. How did this lead to a career in anaesthetics?

I grew up in the home of academic anaesthesia, Madison, where I knew Ralph Waters as a neighbour, and my family spent most summer vacations with anaesthesiologist Philip Woodbridge. The electrophrenic respirator also provided many anaesthesia faculty connections. There were five or six different programmes I wanted to visit, one of which was at Penn. When I visited, the chairman of the department, Robert Dripps, suggested that if biophysics interested me, then I should follow a career in anaesthesia. ‘For example,’ he said, ‘anaesthesia involves monitoring, and at present we don’t have anything that is safe because we use explosive anaesthetic agents. We have to somehow provide monitoring equipment that won’t cause sparks.’ Well, low voltage equipment would do the trick, and I had utilised this with my electrophrenic respirator, so within 5 minutes I had been persuaded to follow a career in anaesthetics.

## Q. This was nearly a short-lived career in anaesthesia, as I believe you had a run in with some Succinylcholine?

During my first 6 months at Penn with Peter Safar, we happened to hear that Dr Dripps was going to receive a shipment of a new drug called Succinylcholine. On the day it arrived, I said to Peter, ‘I would be very interested in actually trying this ourselves, just to see what this very fast-acting drug is like’. I opted to be the subject and Peter gave me what we thought was a tiny dose—way too little to really paralyse me. Well, I lay down on an operating table and Peter gave me 20 mg of Succinylcholine intravenously. The first thing I knew was that I could not say anything and I could not breathe. I did not know how to let Peter know, or what I was going to do about it. Well, my arm was still working so I tried to reach over to the anaesthesia machine and lift up the mask to my face. As my arm collapsed, Peter saw what was happening and ventilated me until I recovered. And that was the good part; due to the muscle contractions, I ached for a week afterwards!

## Q. But fortunately the research continued?

Yes, later I measured and published the rate of uptake of nitrous oxide at the start of anaesthesia in patients.

Dr Dripps then sent me for a year of research in respiratory physiology with Julius Comroe. He asked me to find whether papaverine stimulated breathing via the carotid body. Although I found it did not, because intra-arterial injection just upstream from the carotid body took 15 seconds to cause hyperventilation, Comroe did not believe me so he showed that if he denervated the carotid body, the ventilatory effect disappeared—lesson to me!

A year later, the doctor draft sent me to the National Institute of Health as a US Public Health officer as chief of anaesthesia research in the new Clinical Centre in Bethesda, MD. For use in surgical hypothermia, I published a battery-driven monitor “Telecor” from a single oesophageal probe providing the anaesthesiologist with heart and breathing sounds, patient temperature, and an audible peep with cardiac R waves.

## Q. It was in these early years that you came across the CO_2_ electrode?

In 1953, at the NIH, I went to a meeting of the American Physiological Society in Madison. I heard Richard Stow talk about his idea of making a CO_2_ electrode. He had gone to the library and found out about single electrode measurement for substances like sodium, and was left wondering, ‘If I could measure the pH of some water that was in contact with the blood, perhaps I could measure the PCO_2_.’ So he built an electrode by wrapping a finger cuff from a very thin rubber glove over the end of a pH electrode, having put a little distilled water in between the two. And he found it worked, but said, ‘I couldn’t make it stable as it always drifted too much.’ In the question period which followed, I asked, ‘Did you consider adding sodium bicarbonate to the water?’

‘No, of course not,’ he replied. ‘Bicarbonate is a buffer, and if you put bicarbonate in it, there would be no pH change in the water because it’s a buffer’.

Now I had been working with CO_2_ for years and thought otherwise, so I said, ‘I would like to test that out. Is it OK with you if I go home and try it out?’

Richard replied, ‘Try it, but it won’t work because it’s a buffer’.

Well, within a couple of days I had made a functioning CO_2_ electrode, and to my delight, found that my signal was twice as big as his signal when you changed the PCO_2_. So I wrote to Richard and said ‘Well it works. Why don’t we patent this?’

He said, ‘Well, I don’t want to patent it because it would take too much time away from my patients’.

He did not even publish except for that little abstract that he wrote before he had given his talk to us. I guess both of us underestimated the impact it would have on healthcare, or the importance of publishing early. And I, as a government employee, could not patent it.

## Q. So did this lead to the invention of the blood gas analyser?

In 1956, I organised a meeting at the FASEB (Federation of American Societies for Experimental Biology), and invited a number of people who had been trying to measure PO_2_, oxygen pressure in blood. During this meeting, Leyland Clark, a biochemist from Ohio, showed his invention saying, ‘This is an oxygen electrode’. It had a platinum tip and a reference electrode covered with a thin polyethylene membrane. He had patented the idea and sold it to Beckman. However, he was a biochemist and had no idea how to make it useful for clinical medicine. I seized upon the opportunity. It turned out you had to stir the blood vigorously to get it to work, so my first blood gas machine had a stirring paddle in front of the oxygen electrode. We then added the Clark electrode to the CO_2_ electrode in a water bath, making the first PO_2_ and PCO_2_ blood gas analyser.

## Q. After Penn, life took you to San Francisco?

Yes, after three years of anaesthesia research at NIH and a second year of anaesthesia training with Stuart Cullen at the University of Iowa, I and my family of three children moved to San Francisco at Julius Comroe’s invitation to found both the Department of Anaesthesia, and a Cardiovascular Research Institute at UCSF. I bought a house there within a matter of a few weeks in Ross, a Marin county suburb, and I am still living in it 58 years later!

And there I had an agreement that I would have 4 days a week in research and 1 day a week in clinical. That was wonderful.

## Q. Did you ever think to patent the idea, or set up a company to make the CO_2_ electrodes or this initial blood gas analyser?

Yes, but I was far too busy with research and uninterested in becoming a manufacturer. And even looking back, I am happy the way it went.

One day, Forrest Bird, who had been making respirators, was visiting our operating rooms, and learned about my home-made blood gas analyser and offered to manufacture the CO_2_ electrodes with his company, The National Welding Company. As it seemed like a good idea, his company bought commercial pH electrodes, incorporated them with a little plastic device that held the membrane and a reference electrode, and sold them as the ‘Severinghaus CO_2_ Electrode’. Well the first thing I said was, ‘Take my name off of it. It’s a CO_2_ electrode. It’s not mine, and Richard Stow was the inventor’. So what did they do? They gave me one of them without my name on it, and sold all the rest with my name still on it!

## Q. And the blood gas machine?

Well several people started trying to make them, including a company called Radiometer in Copenhagen. I worked with them (with frequent visits) and eventually we got it right, realising that you had to warm the blood up to body temperature before inserting it into the electrodes. Radiometer has become the manufacturer of the world’s best blood gas analysers.

## Q. Did you enjoy your one-day-a-week of clinical?

Oh yes I loved it. Teaching was a lot of fun and I had special interest, because as a teacher I could introduce a student to something they had never done before. They enjoyed it, and so did I.

## Q. Did you miss clinical work when you retired?

Well no. I am happy with what I did. I still give three lectures to the residents every year, the history of blood gas analysis, a high altitude human research story, and acid-base balance. I always think every year will be the last year but they invite me back again. Apparently, they are popular.

## Q. With regards to your research at UCSF, there really are huge amounts we could talk about. What stands out?

Well there were two main things. Firstly, I had to improve the blood gas analyser by adding a pH electrode. I had only put O_2_ and CO_2_ electrodes in the one I published in 1958, but as soon as I got to San Francisco, I added an internal pH electrode and that is what really made what is called a blood gas analyser. The original is now housed at the Smithsonian Museum, and this is what Radiometer (and many other companies) went on to build.

Secondly, fortunately, internist Robert Mitchell joined my lab. He had treated 2 COPD very hypercapnic patients by injecting mildly acidified artificial CSF into their lumbar spines and saw their PCO_2_ fall from the 80s to normal in an hour. He and visiting German physiologist Hans Loeschcke then located the brain CO_2_ chemoreceptors (on the ventral medullary surface). As I had this portable device for measuring blood gases, we thought we could find whether acclimatisation to high altitude depends on changes in spinal fluid and arterial PO_2_ and PCO_2_. So off we went to study acclimatisation to high altitude at the University of California White Mountain laboratories. That began my work in high altitude physiology. Other studies over time included repeating these acclimatisation studies in Peruvian and Bolivian high-altitude natives. We also investigated the control of cerebral blood flow (CBF) at altitude, joined by Tom Hornbein soon after his ascent of Everest.

## Q. Amongst the many years of research, you also undertook four sabbaticals: Copenhagen twice, Nijmegen, and Oxford.

Yes, in the 1950’s three Scandanavian physiologists formed a society interested in control of cerebral blood flow. It still exists as The International Society of Cerebral Blood Flow and Metabolism. I was working in Copenhagen with Niels Lassen to learn whether PCO_2_ alters brain blood flow by arteriolar or tissue PCO_2_. This involved taking blood samples from the internal jugular vein by direct needle puncture. Niels was an expert. However, on the occasion when I was the subject he had trouble with my anatomy, and eventually hit a nerve that paralysed the right side of my face and tongue. Fortunately, the nerve conduction came back about two days later.

## Q. Another part of your research relates to developing the first ever remote monitoring in operating rooms. How did that come about?

We had an operating suite where I was working as an anaesthesiologist, with ten operating rooms all in a kind of a circle. In 1975, I acquired an unused mass spectrometer used for anaesthetic research by Ted Eger, so I thought we could try to set it up so that it could continuously measure the expired gas concentrations of anaesthetic, oxygen, and CO_2_ in a patient during surgery. At night, when nobody was looking, we strung small nylon catheters above the roof tiles from each of the ten operating rooms to a central area where we set up this system with the mass spectrometer. The system was put together by faculty member Gerald Ozanne, myself, and a brilliant technician, Bill Young, who designed and wrote the programmes for the operating system. My electronic physicist son Ed built a valve device that switched the gases going into the mass spectrometer from one room to the next by a whole series of little valves. This mass spectrometer could provide information that was then sent back to each theatre on a dumb terminal screen. It displayed data as numbers or plots about once a minute, typically of both the PCO_2_ and the anaesthetic depth. The residents, faculty, and nurse anaesthetists in the operating rooms loved it. The faculty could sit in their office and find out what was going on in the operating room that they were responsible for. They could watch the anaesthetic and N_2_0 concentrations, PO_2_, and PCO_2_, and send and receive notes to any operating room from their office.

That was the first multi-room method of gas monitoring to be established worldwide, which as you know, has now turned out to be extremely popular. Additionally, different theatres could send notes to each other or to anyone from these terminals using Arpanet, established long before the World Wide Web and emails.

## Q. This gas monitoring led on to the development of the concept of minimum alveolar concentration (MAC) for anaesthetic agents?

No, MAC came a decade earlier, in 1968, from a lunchtime group discussion with Ted Eger. He had noticed that the end expired concentration of an anaesthetic is a very good measure of the depth of anaesthesia. He proposed determining what concentration is just enough to keep a patient from moving when the surgeon starts cutting. It occurred to me to call that MAC-1. MAC (Minimal Alveolar Concentration) would be the ratio of end tidal value in a patient to MAC-1. Why MAC? I knew that in aviation, MACH is the ratio of the speed of a plane relative to the speed of sound.

## Q. A large proportion of your work has also involved the development of pulse oximeter testing?

Yes. That followed my transcutaneous oxygen measurement work—a system that has been all but wiped out because of the invention of pulse oximetry. I got involved as I was invited to write the history of it for the first world meeting on pulse oximetry in 1985. So I looked at the literature to find out who invented it and how it came about. And do you know, there was not one mention of the inventor in the literature. After much searching, I was finally introduced to Takuo Aoyagi—the inventor. I then rewrote and republished the real story.

## Q. So on the back of that meeting, I believe you started up a laboratory for testing the accuracy of pulse oximeters?

The manufacturers and I wanted to know how accurate pulse oximeters were at low saturations. In 1986, I got permission from the committee on human experimentation at UCSF to take volunteers down to an oxygen saturation as low as 40 % just long enough to obtain an arterial sample. My credentials showed that I had been doing similar studies at high altitude looking at respiratory control, and I had publishing altitude studies for over 20 years. This permission is reviewed yearly and still stands. The manufacturers were later told by the FDA that we do not need to provide any information below 70 %. That lab is still going strong although I am no longer involved.

## Q. Aside from the lab, what is keeping you busy now?

I was invited to write the history of the discovery of oxygen in 1993 for a Penn State University Medical School Anaesthesia Department lecture. I learned about Carl Scheele and realised that I really knew very little about the discovery of oxygen and how it happened. Ever since then, I have gradually rediscovered my (and everyone’s) ignorance of many scientists who over eight centuries had discovered or participated in discovering oxygen’s history. It is keeping me busy, as are conferences and lectures.

## Q. I would like to finish with a few generic questions to which you may, or may not have answers. What would you describe as the most seminal achievement of your career?

Initiating the field of electronics in monitoring during anaesthesia.

## Q. Looking back, you have published over 470 papers or chapters and multiple books. Is one more memorable than the rest?

Well, I think the first blood gas analyser using my CO_2_ electrode is the most memorable paper I have ever written. The National Library of Medicine told me in the early 1960s it was among the most often quoted of my papers. Now that commercial blood gas analysers are copied worldwide, there is no interest in reading about it!

## Q. If I was to give you a fully-funded lab, and all the time in the world, what are the questions you would go to answer now?

I have absolutely no idea.

## Q. Obviously a number of people have influenced your career. Do any particular people stand out as key influences?

My father supported me into working on things, not thoughts. At Haverford College, physics professor Dick Sutton illustrated how fascinating demonstrations are the best lectures. His teaching switched me from majoring in chemistry to physics. Bob Dripps persuaded me to go into anaesthesia, Roy Vandam at Penn taught me how to write precisely and carefully. Julius Comroe, a careful experimenter, a wonderful leader, and an extremely good speaker using really good jokes, suddenly devised a method to get me to UCSF.

## Q. And finally, if you could sit down and have a chat with anyone in history, who would that be?

Well perhaps Priestley. He led the split among liberal Christians to Unitarianism, was a great supporter of liberalising education contrary to the public opinion of the time, and was a self-taught, multi-talented scientist. However, he was unable to abandon the phlogiston theory even twenty years after everybody else gave it up because he had called oxygen ‘dephlogesticated air’, and if he gave up phlogiston, he must have thought it would be giving up his discovery!

